# Multi-scale modelling predicts plant stem bending behaviour in response to wind to inform lodging resistance

**DOI:** 10.1098/rsos.221410

**Published:** 2023-01-04

**Authors:** Tarun Gangwar, Alexander Q. Susko, Svetlana Baranova, Michele Guala, Kevin P. Smith, D. Jo Heuschele

**Affiliations:** ^1^ Department of Civil, Environmental, and Geo-Engineering, University of Minnesota, Twin Cities, MN, USA; ^2^ Agronomy and Plant Genetics, University of Minnesota, Twin Cities, MN, USA; ^3^ Plant Science Research Unit, USDA – Agricultural Research Services, St. Paul, MN, USA

**Keywords:** finite-element method, multi-scale material model, oat, wheat, wind, lodging

## Abstract

Lodging impedes the successful cultivation of cereal crops. Complex anatomy, morphology and environmental interactions make identifying reliable and measurable traits for breeding challenging. Therefore, we present a unique collaboration among disciplines for plant science, modelling and simulations, and experimental fluid dynamics in a broader context of breeding lodging resilient wheat and oat. We ran comprehensive wind tunnel experiments to quantify the stem bending behaviour of both cereals under controlled aerodynamic conditions. Measured phenotypes from experiments concluded that the wheat stems response is stiffer than the oat. However, these observations did not in themselves establish causal relationships of this observed behaviour with the physical traits of the plants. To further investigate we created an independent finite-element simulation framework integrating our recently developed multi-scale material modelling approach to predict the mechanical response of wheat and oat stems. All the input parameters including chemical composition, tissue characteristics and plant morphology have a strong physiological meaning in the hierarchical organization of plants, and the framework is free from empirical parameter tuning. This feature of our simulation framework reveals the multi-scale origin of the observed wide differences in the stem strength of both cereals that would not have been possible with purely experimental approach.

## 1. Introduction

Lodging, the failure of a plant to fully recover after bending, occurs when an extreme force from wind or rain is applied to the plant. Lodging is particularly detrimental to the cultivation of cool-season cereals such as *Avena sativa* L. (oat) and *Triticum aestivum* L. (wheat) by causing substantial yield reductions [1] from reduced photosynthesis and difficulties in mechanical harvest [2]. There are two major classifications of lodging. The first is root lodging, which occurs when the roots fail to be anchored to the soil substrate by either root slippage or root breakage. The second is stem breakage, which constitutes buckling along any point on the stem.

Plant breeders use field-based visual severity ratings for both types of lodging to directly select breeding lines that are lodging resistant or to identify genes and linked markers to indirectly select for lodging resistance. However, field ratings of lodging characterize the end result, but not the plant’s direct interaction with the wind [3]. Plant–wind interactions encompass many physical plant parameters whose optimization, in theory, could produce a more lodging-resistant cereal [4]. Superior ideotypes have been proposed that combine parameters, such as a lower centre of gravity on the stem with increased stem diameters, to improve plant wind resistance and reduce the probability of lodging [5,6]. Engineering models looking solely at plant structure have also been created to predict lodging-resistant ideotypes [7–9]. These models incorporate stem structural scale components such as diameter, cross-section characteristics and node characteristics. Coordinated simulation-based research and experimental studies under a controlled wind environment to understand the aerodynamic and bending behaviour of cereal stems could provide new insights to breed for lodging resistance.

Wind tunnel testing of cereal crops offers the opportunity to obtain detailed phenotypes relevant to lodging resistance that cannot be quantified under typical field conditions. Wind tunnel testing serves to isolate two aspects of the complicated problem of lodging in cereals: the estimation of wind-induced forces on the plant that causes stem bending, and the response of the stems under a known drag force. Wind tunnel research on cereal crops has focused on the canopy level to calculate the fluctuating airflow at canopy height [10]. Also, portable wind tunnels have been used to manipulate airflow *in situ* for wheat, barley and maize [11–13]. However, these experiments focused on selecting lodging-resistant plants and not identifying traits that contribute to lodging-resistant ideotypes.

One major challenge is collecting and analysing data from experiments that capture plant stem behaviour in environments that produce lodging. Recently, increased video analysis capabilities combining advanced computer vision and automated image processing tools have been used to quantify the frequency and amplitude of plant movement in field environments [14]. Controlled wind tunnel testing supported by advanced video analysis methods can rapidly quantify the aerodynamic and bending behaviour of cereal stems with the help of metrics such as plant drag coefficients and the stem deformation profile. Correlations of these behaviour metrics with measured plant phenotypes will help identify plant traits that affect stem bending behaviour.

Often stand-alone experimental studies tend to be unreliable and inconclusive in correlating traits with lodging behaviour. Reasons include the complex anatomy and morphology of crops at multiple length scales, the complicated interaction of the traits that contribute to lodging and environmental factors and forcing uncertainties. This conundrum was summarized by Garber & Olson [15] almost 100 years ago as *lodging in cereals is dependent on so many factors of unequal value in the different sorts that no one factor seems to be correlated closely enough with lodging to be of much value as a selection index.* Considering all these traits and their interactions in an experimental study will be prohibitively costly in time and labour. By contrast to empirical experiments, computer simulations or so-called *in silico* experiments, can integrate many complex factors and explore a large parameter space to understand lodging behaviour [16]. In a recent perspective note, Benes *et al.* [17] has emphasized the role of multi-scale computational model based *in silico* experiments for crop improvement strategies. However, these simulations require massive simplification to translate nature’s complexities into mathematical formulations, and, therefore, the conclusions from these simulations must bear these simplifications in mind. Although computer simulations cannot replace experimental studies, simulations in conjunction with controlled environment testing could improve the design of experimental studies for lodging.

Plant materials organize themselves hierarchically across multiple length scales that range from base constituents such as lignin, cellulose, hemicellulose, pectin to cell wall, functional tissue, organ and whole plant levels [18,19]. Brulé *et al*. [20] reviewed the effect of genetic modification on the stiffness and strength of the genetic model plant *Arabidopsis thaliana*. In their study, a consistent explanation of their findings was not possible as the genetic modifications affected the plant structure and the physiological response at multiple levels. The authors concluded: *What is needed is a comprehensive, systematic and consistent multiscale mechanical analysis of structural parameters across length scales to feed into an integrated model of the development of plant stiffness.* Therefore, an essential prerequisite for the efficient and reliable prediction of lodging resistance of cereals is to accurately relate their mechanical behaviour to their composition and quantifiable morphological traits, while considering the underlying multi-scale physics.

We recently developed and validated a multi-scale material model to accurately predict macroscale stiffness and strength properties of stem materials from their hierarchical microstructure [21,22]. All model parameters can be exclusively obtained from chemical analysis and micro-imaging data on cereal stems without any phenomenological tuning. This material model can be integrated into a finite-element framework to simulate the mechanical behaviour of cereals under controlled experimental settings. These simulations can relate the stem strength behaviour with plant traits quantitatively and, therefore, lead to important insights to improve lodging resistance of cereals. We note that, in the past decade, the multi-scale material-model-based finite-element simulations have contributed significantly in understanding and developing patient-specific diagnostic tools for bone diseases [23–27].

In this study, we attempt to characterize lodging behaviour of wheat and oat using both wind tunnel experimental observations and multi-scale material-model-based finite-element simulations. Specifically, this study seeks to determine whether model-based predictions of cereal stem bending fall within the observations of independently assessed cereals in a wind tunnel, which encompass a range of lodging susceptibilities. We establish that the novel combination of both approaches reveals the multi-scale origin of failure mechanisms leading to lodging that would not have been possible using these approaches independently. Our objectives are (i) to quantify the stem bending and failure behaviour of oat and wheat plants in a wind tunnel, (ii) to build and validate an independent finite-element simulation framework integrating existing multi-scale material modelling approach to predict the stem strength behaviour of single oat and wheat stems, and (iii) to use the framework for understanding the causal relationship of the observed stem behaviour in the wind tunnel with the physical traits at different length scales that could confer lodging resistance for these major cereal crops.

## 2. Methods

### 2.1. Wind tunnel experimental set-up and data acquisition

The experimental component of this work consists of a multi-step procedure devoted to quantifying mechanical and physiological properties of select cultivars before and during aerodynamic tests in the wind tunnel, and identifying a parameter space for comparison with numerical simulations.

*Wind tunnel germplasm:* We tested eight cultivars with up to three replicates each of two cereal crops (oat and wheat), selected based on their morphological variability and their variable lodging resistance in field trials (table 1). Plants were grown in a greenhouse until approximately 18 days after all completed heading, with one experimental block (location on greenhouse bench) tested in the wind tunnel per day. Plants were seeded into 16.5 cm diameter, 13 cm tall terracotta pots in the greenhouse in January 2018 containing peat-based LC8-Sunshine mix (Sun Gro Horticulture, Agawam, MA, USA). The potted seedlings were randomly assigned to one of three blocks on the greenhouse bench in a randomized complete block design. Greenhouse temperatures were maintained at 21°C during the day and 18°C at night, with an 18-h photoperiod. Plants were removed from the greenhouse on the day of their wind tunnel testing at approximately 18 days after heading, with one block tested in the wind tunnel per day. The media mix in each pot was moistened and consistently kept at field capacity in the time leading up to the wind tunnel testing.

**Table 1.** Germplasm used in the wind tunnel.

latin name	crop	cultivars
*Avena sativa* L.	oat	‘Gopher’, IL078721, ND021052, ‘Reins’
*Triticum aestevium* L.	wheat	‘Linkert’, MN113946, ‘Rollag’, ‘Shelly’

*Physical plant measurements before wind tunnel tests:* Prior to wind tunnel testing, we took several measurements of plant morphological traits. Heading date (anthesis) was recorded in the greenhouse as the number of days since planting when 50% of the first panicle or spike emerged. Whole plant strength was estimated using a load cell mounted to an aluminium bar the day before subjecting each block to wind tunnel testing, measuring the force *F*_*s*_ required (in N) to bend all stems at the half-height point in one pot to an approximate 50° angle with respect to the ground [28]. A detailed description of this method can be found in [29]. Interested readers can also refer to Cook *et al.* [30], Robertson *et al.* [31] and Spink *et al.* [32] for alternative approaches. The $50^{\circ }$ bending angle is hereafter referred to as the reference stem deformation. Plant height, defined from the base of plant stems to the tip of the tallest panicle or spike (in cm), was measured the day before wind tunnel testing. Following the experiment, we destructively sampled each plant’s stem and leaf tissue (without panicles or spikes) and weighed them collectively following 3 days in a 60°C dryer to obtain an estimate of leaf and stem biomass (gram).

*Wind tunnel specifications:* We subjected each pot to controlled airflows in the atmospheric wind tunnel of the St. Anthony Falls Laboratory at the University of Minnesota Twin-Cities. The wind tunnel, equipped with a 149 kW fan, is a 37.5 m long closed-loop, with a test section of 16 m in length and 1.7 × 1.7 m in cross-sectional area (for additional details, see [33]). All wind tunnel tests completed in this work consisted of a single plant exposed to an undisturbed wind with a uniform velocity profile, above a shallow canonical logarithmic boundary layer not exceeding 0.2 m height [34]. The plant pots were flush-mounted with the tunnel surface at the end of the test section using 9.5 mm thick aluminium sheet metal with a hole matching the pot diameter and an underneath wooden support structure. Wind tunnel mean velocity was measured at a fixed height of 60 cm above the wind tunnel floor using a Pitot tube recording dynamic pressure and a thermocouple recording air temperature, required for air density estimate. Pitot tube data were averaged over a 1 s window and used to calculate the wind velocity at every second of the video.

Each plant was exposed to constant wind velocities of 4, 8 and 12 m s^−1^ (corresponding to 140, 270 and 400 r.p.m. fan speed, respectively) at consecutive 50 s intervals. The wind velocity *v*_*w*_ was accelerated between intervals and decelerated at the end of the 12 m s^−1^ test down to 4 m s^−1^ over a 5 s interval; then it was stopped at 2 min 50 s into the test. Camera recording continued until 3 min 5 s after test initiation, recording approximately 35 s of plant recovery as the wind in the tunnel decelerated and stopped. The wind tunnel testing protocol consisted of an uninterrupted video of plant bending lasting 3 min 5 s and covering the entire sequence of mean velocity variation. Videos of each wind tunnel test were captured with a Canon EOS 5D camera with an Ultrasonic EFS 60 mm lens with a maximum aperture of f/1.5. Each video has a spatial resolution of 1920 × 1080 pixels and a temporal resolution of 24 frames per second. Measurements of plant deformation extracted from each video recording frame were averaged over 24 frames to align temporally with the pitot tube measurements averaged over the same 1 s time interval. During field-based wind tunnel experiments wind speeds of 12 m s^−1^ resulted in maximum lodging variation for screening purposes [11,12], while bending reference of 50° was chosen due to the maximum strength exhibited experimentally when measured at half height [35].

**Figure 1.**
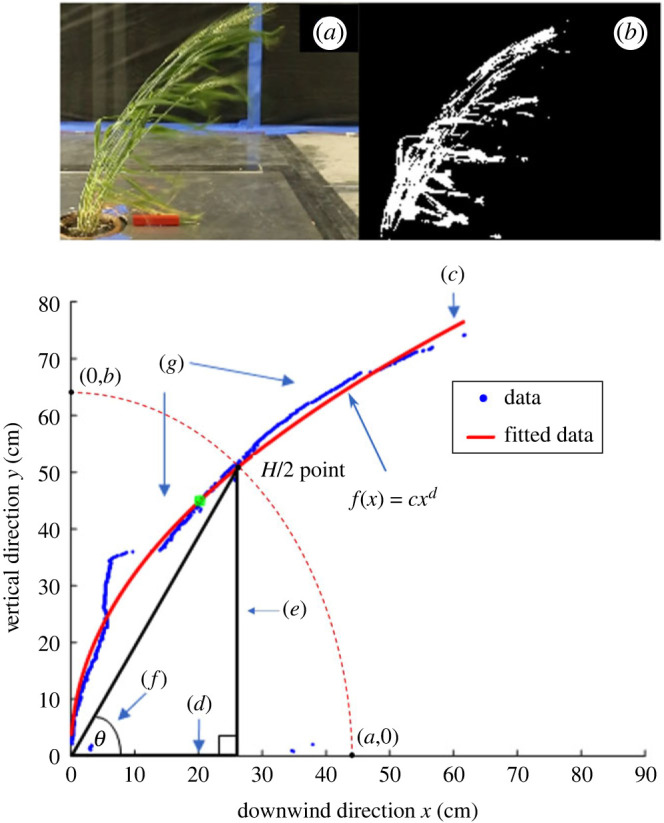
Example of video-derived phenotypes for each frame. (*a*) Unmasked frame, (*b*) masked plant, (*c*) windward edge with fitted power curve, (*d*) fixed downstream distance *x*_0_ where half length of stem (*H*/2) defines a 50° angle with wind tunnel floor, (*e*) vertical elevation *f*(*x*_0_) of the bent stem at the half length point, whose intersection at *H*/2 point is used to estimate the CL_*r*_ curve (dashed), (*f*) bending angle *θ* at any frame and (*g*) extracted and filtered stem bending profile.

*Plant measurements during wind tunnel tests:* Several phenotypes used to describe plant bending were obtained from the captured videos. Briefly, each frame of a video (figure 1*a*) was first masked to show plant tissue (figure 1*b*). The masked points on the windward edge of the plant were then plotted and fit by a power law curve *f*(*x*) = *cx*^*d*^ (figure 1*c*) defined by a scaling coefficient (*c*) and a power law exponent (*d*). The fitted curve for each plant was used to estimate the bending angle *θ* at any instant during the test (figure 1*e*). A sample video (CLrVideo.avi) is also provided in electronic supplementary material, text S1.1 that illustrates the steps visualized in figure 1.

The drag coefficient *C*_*d*_ was quantified at the reference deformation, i.e. *θ* = 50°. We denote *F*_*w*_ as the equivalent horizontal force experienced in the wind tunnel, *v*_*w*_ and *ρ*_air_ as the velocity and density of the air, and *A* as the effective frontal area, that is the projection of the plant area exposed to the airflow along the windward edge, at the reference deformation. Then, the drag coefficient *C*_*d*_ follows as

2.1 $$C_{d}=\frac{2F_{w}}{\rho_{\mathrm{air}}Av_{w}^{2}}.$$

Before the aerodynamic tests, we measured the force *F*_*s*_ required to bend all stems to this angle from the load cell measurements applying a follower force at the half-height point. In electronic supplementary material, text S1.3, we derive the force *F*_*w*_ as a function of the measured force *F*_*s*_ using the principle of virtual work. It was not possible to place the camera along the windward edge in the current wind tunnel set-up, and, therefore, the effective frontal area *A* cannot be measured directly. Instead, following electronic supplementary material, text S1.2, we estimated the upper bound on the frontal area *A*_*ub*_ using recorded videos of plant deformation in the streamwise vertical plane. Replacing *A* with *A*_*ub*_ in (2.1), the lower bounds on the drag coefficient *C*_*d*_ is estimated. The velocity *v*_*w*_ and air density *ρ*_air_ were obtained at the reference deformation from the pitot tube time history, as detailed in electronic supplementary material, (S.17)–(S.19) and text S1.4. The subsequent range of Reynolds number values, based on the incoming velocity, plant diameter and kinematic viscosity of air (*R*_*e*_ = *v*_*w*_ *D*/*ν*) ranged between 2.7 × 10^5^ and 1.59 × 10^6^.

We quantified stem bending resistance under the action of wind drag using a second metric based on the coefficient of lodging resistance (CL_*r*_) introduced by [36] (electronic supplementary material, figure S3i–ii). CL_*r*_ is a proportional measure of the amount of torque resisted by a cereal stem during bending under a known force: its estimate is provided for the stem in equilibrium at the moment of known reference deformation (*θ* = 50°) and associated force *F*_*w*_ , i.e. at the same instant in the video where the drag coefficient is estimated. Equations electronic supplementary material, (S.23)–(S.28) describe the estimation of CL_*r*_ from videos and could be found in electronic supplementary material, text S1.5. We note that the plants that did not reach the reference deformation corresponding to a *θ* = 50° bending angle during the test, the minimum bending angle *θ* reached recorded and the associated pitot tube velocity were used to estimate the drag coefficient *C*_*d*_ and the coefficient of lodging resistance CL_*r*_.

*Data statistical analysis:* We analysed the crop, cultivar nested within crop, and block (position on the greenhouse bench) effects in an additive linear model of the video-derived phenotypes using an analysis of variance (ANOVA) on *C*_*d*_, *θ*_max_, *d* and CL_*r*_ response values using the linear model

2.2 $$y_{ijk}=\beta_{0}1+\beta_{1}x_{i1}+\beta_{2}x_{i(j)2}+\beta_{3}x_{k1}+\varepsilon_{ijk}$$

with *β*_1_ representing the effect of the *i*th crop, *β*_2_ is the effect of the *j*th cultivar nested within the *i*th crop, *β*_3_ the effect of the *k*th block and $\varepsilon_{i}jk$ is the error term. For crop effects, we present the mean separations according to a least significant difference (LSD) test with a threshold value for *p* < 0.05. We calculated Pearson correlation coefficients among the linear relationships of the physical and video-derived plant phenotypes using the cultivar averaged values.

### 2.2. Multi-scale material-model-based finite-element simulations of oat and wheat stems

**Figure 2.**
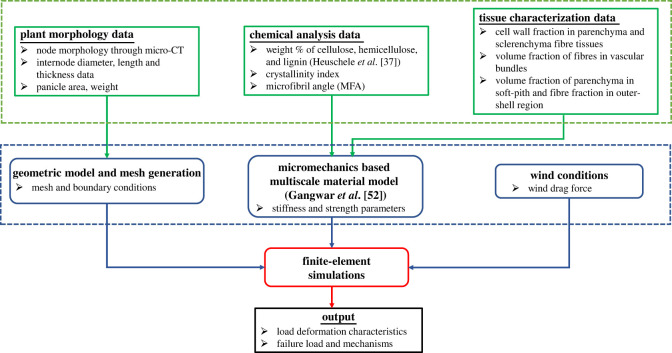
Conceptual overview of multi-scale material-model-based finite-element simulation framework. The green boxes indicate input data required for the model. Blue boxes denote the existing essential tools required to generate material and geometric properties for simulations with external applied wind drag force. Outputs are outlined in black.

Figure 2 outlines the conceptual overview of the multi-scale material-model-based finite-element simulation framework of single cereal stems under given wind conditions. In this figure, the green box summarizes all the input data required for the simulations. These data comprehensively characterize the single stem physiology that consists of (i) the constituent materials’ chemical composition, (ii) cellular structure and tissue characterization, and (iii) plant morphology, including node, internode and panicle characteristics. The blue box contains the preprocessing steps required for the finite-element simulations, including (i) the generation of geometric model and mesh, (ii) macroscale stiffness and strength properties, and (iii) applied external wind drag force. Combining all these inputs, we build a finite-element model for wheat and oat stem in the commercial software ABAQUS (red box). Later, simulation outputs (black box) are analysed in conjunction with wind tunnel experiment results for insights on the multi-scale origin of lodging resistance of wheat and oat. The essential components of this framework are discussed below.

*Evidence-based multi-scale material model for cereal crops:* We recently derived a micro-mechanical model for the effective stiffness and limit strength of crop stem materials from their hierarchical structure in the framework of continuum micromechanics [21]. This material model is independent of any experimental setting-based phenomenological tuning. The model was extensively validated previously against the four-point bending flexural test performed on oat stems. Micro-CT images of stems before and after flexural tests were taken by Minnesota Dental Research Center for Biomaterials and Biomechanics (MDRCBB) to determine micro-scale failure points. Electronic supplementary material, figure S4 illustrates the conceptual overview of our model, parametrized in terms of the properties of the base constituents as well as morphology and volume fractions of all heterogeneous components at each level of the hierarchical structure of cereal stems. Parameter inputs to this model are retrieved from chemical analysis and imaging data at different length scales, obtained from light and transmission electron microscopy (see samples shown in electronic supplementary material, figure S5). We determined these parameters in our earlier works [22,37]. Electronic supplementary material, text S2 summarizes all the required input data and calculated stiffness and strength parameters for oat and wheat stems.

**Figure 3.**
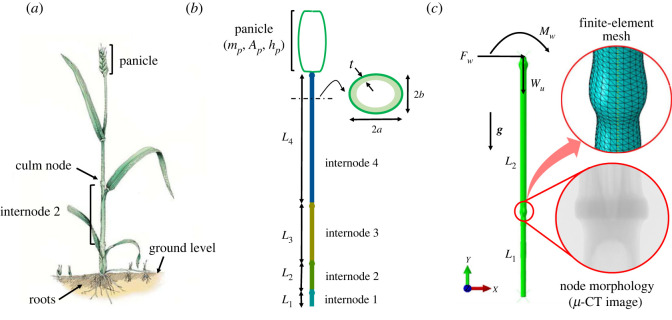
(*a*) Typical anatomy of a cereal plant, (*b*) macroscale geometry of the plant denoting measured plant morphology traits and (*c*) geometric model using node morphology from micro-CT data for finite-element simulations with applied external forces. Micro-CT image provided by MDRCBB.

*Geometric model and wind-induced drag force*: Figure 3 illustrates a typical cereal plant anatomy and its schematic description with measured plant morphology data, including length of internodes, cross-sectional characteristics and panicle characteristics. The data are compiled in electronic supplementary material, tables S3 and S4. To create a geometric model for finite-element simulations, we only include two lowermost internodes (figure 3*c*), given that stem failure has been generally observed in this region. Node morphology in the model is derived from micro-CT data detailed in [21]. We further assume that oat and wheat internodes have a hollow circular cross-section with an outer radius as average of the radius of the corresponding section.

The rest of the upper plant exerts an external gravitational force equal to its weight *W*_*u*_, which is calculated as

2.3 $$W_{u}=g\left(m_{p}+\rho \pi \sum_{i=3}^{4}L_{i}t_{i}(2r_{i}-t_{i})\right),$$

where *g* is the gravity acceleration, *m*_*p*_ is the measured mass of panicle, *ρ* is the density of stem and assumed to be equal to 3 × 10^−4^ g mm^−3^ for both oat and wheat, *L*_*i*_ *t*_*i*_ and *r*_*i*_ are the length, thickness and average cross-section outer radius of the corresponding section *i*, respectively. In addition, the weight of the modelled part is introduced as a body force *G* = *ρg* (figure 3*c*). We assume that only the panicle area contributes significantly and neglect the stem area for the simulations. This assumption is motivated by the fact that the stem area is reasonably smaller than the panicle area and the force exerted on the lower part of the stem is negligible due to the boundary layer effects. This force is approximated via force *F*_*w*,0_ and moment *M*_*w*,0_ defined as

2.4 $$F_{w,0}=\frac{1}{2}\rho_{a}A_{\;p}C_{d,p}v_{w}^{2}$$

and

2.5 $$M_{w,0}=F_{w,0}\left(L_{3}+L_{4}+\frac{1}{2}h_{p}\right),$$

where *ρ*_*a*_ is air density as $1.225\times 10^{-6}\;g\;{\mathrm{mm}}^{-3}$, *A*_*p*_ is the panicle frontal area, *C*_*d*,*p*_ is the drag coefficient characterizing the aerodynamic characteristic of the panicle, *v*_*w*_ is the wind velocity, *L*_3_ and *L*_4_ are lengths of third and fourth internodes and *h*_*p*_ is the panicle height. The parenthesis term in (2.5) is essentially the lever-arm of force *F*_*w*,0_ causing moment *M*_*w*,0_.

*Finite-element models for wheat and oat stems:* We build a finite-element model for each wheat and oat stem in the commercial software ABAQUS, discretizing the inner solid core region with 20-node brick elements (C3D20R) and the outer-shell region with eight-node shell elements (S8R), respectively. Since the basis functions of the shell conform with those of the solids at the coupling surface, surface locking between the shell and solid elements is prevented [38]. We assign the macroscopic stiffness moduli and strength properties for both the solid-pith and shell region from electronic supplementary material, tables S5 and S6. The bottom nodes of the model are fixed. Load conditions include body force *G*, applied through the whole model, and external forces *F*_*w*_ , *W*_*u*_ and moment *M*_*w*_ , applied to the upper node located at the centre of the upper cross-section of the specimen. To match wind tunnel forcing, in the absence of atmospheric unsteadiness, we assume quasi-static conditions. Therefore, we perform nonlinear finite-element analysis using static-general algorithm in ABAQUS. The model deformation is computed in two steps: in *the loading step*, wind force *F*_*w*,0_ and moment *M*_*w*,0_ are gradually increased, simulating loading due to the wind condition, and in *the unloading step*, they are gradually decreased, simulating the unloading process after wind conditions. The force *W*_*u*_ remains the same during all two steps.

## 3. Results and discussion

To better understand the complex aerodynamic and stem bending behaviour of cereal stems, we first investigated the relative differences of wheat and oat using wind tunnel experiments. Next, we simulated and validated the stem behaviour of both cereals focusing on failure mechanisms using the simulation framework summarized in figure 2. Finally, we explain the underlying physics of the observed deformation and failure behaviour with the help of our simulation framework, which allows to identify the effect of key physical traits such as chemical composition, tissue characteristics and plant morphology that affect cereal stem behaviour under wind stress.

### 3.1. Quantification of wheat and oat stem bending behaviour through wind tunnel measurements

*Aerodynamic behaviour of cereal crops*: At the reference deformation, the lower bound on the cereal drag coefficients (*C*_*d*_) did not vary at the crop (*p* = 0.91), block (*p* = 0.74) or cultivar nested within crop (*p* = 0.71) levels. This indicates that individual plant differences dictate the aerodynamic behaviour of cereal crops instead of the aggregate crop differences of oat versus wheat. The mean reported *C*_*d*_ was 0.72 for wheat and 0.76 for oat (table 2), with the 95% confidence interval ranging from of 0.46 to 0.98 for wheat, and 0.27 to 1.25 for oat. Our *C*_*d*_ estimates for wheat and oat are greater than the previously reported drag coefficient for fountain grass [39], which ranged between 0.46 and 0.37 over similar Reynolds number values (5 × 10^5^ < *R*_*e*_ < 13 × 10^5^) as in our experiment. Our mean cereal *C*_*d*_ values are also higher than those assumed in theoretical studies on canopy airflow by Finnigan & Mulhearn [10,40] and Katul *et al.* [41]. Despite the overestimate of the frontal area *A*, our *C*_*d*_ values are likely to be underestimated, as the force required to bend the plant to the reference deformation was rarely reached in wheat at 12 m s^−1^ maximum wind tunnel velocity speed (see discussion of limitations below). This underestimation would serve to increase the mean wheat *C*_*d*_ closer to that of oat, and well within the 95% confidence intervals for the *C*_*d*_ of both oat and wheat.

**Table 2.** Mean separation for video estimated parameters and physical phenotypes between wheat and oat.

phenotype	oat	wheat
1. mean *C*_*d*_	0.76	0.72
2. mean CL_*r*_	0.05*	0.13
3. mean *θ*_max_ (°)	47.4*	55.1
4. mean *d*	0.63	0.57
5. heading (days)	47.4	47.7
6. plant strength (N)	0.78	1.23
7. height (cm)	98.44*	72.09
8. biomass (g)	13.11*	9.87
9. mean recovery *θ* (°)	4.3	5.19

*indicates significant differences (*p* < 0.05) between means in the same row.

Our definition of drag coefficient relies on (i) a specific estimate of the plant frontal area exposed to uniform wind, thus excluding the effects of the terrain roughness or canopy sublayer and (ii) a specific stem reference deformation at which the force was measured. Thus, we cannot strictly compare with other *C*_*d*_ estimates in the literature. However, the quantification of the drag force is important in the comparative analysis between different crops, in particular for the assessment of the different stem response to comparable aerodynamic forcing, enforced by controlled wind tunnel experiment. Nonetheless the wide confidence intervals around the crop mean *C*_*d*_ in these experiments point to variation in drag coefficients depending on the variability of individual plants. However, we emphasize that the lower bound on the drag coefficient cannot account for the aerodynamic differences caused by the smearing of leaves under wind. For instance, the characteristic differences among crops in reorienting the leaf foliage in the direction of wind would result in wide differences in the frontal area *A*. In this case, the lower bound approach cannot capture this aerodynamic behaviour and will require a direct measurement of the frontal area.

*Variation in stem bending parameters*: In contrast to the drag coefficient, the ratio of the torque resisted to torque applied (coefficient of lodging resistance or CL_*r*_) was significantly higher in wheat compared with oat. Video analysis enabled tracking the transit of a fixed mid-height point along the modelled stem curve to estimate the CL_*r*_ at the moment of known drag force *F*_*w*_. The CL_*r*_ value at the reference deformation varied significantly between oat and wheat (*p* = 0.008) but not among blocks (*p* = 0.41) or among cultivars nested within crops (*p* = 0.45). Wheat had a significantly higher mean CL_*r*_ than oat (table 2). The CL_*r*_ value for wheat, as with *C*_*d*_, is probably an underestimate of the true average CL_*r*_ for these wheat cultivars, as the drag force needed to bend the plant to the reference deformation was not met for 72% of the wheat plants tested at the 12 m s^−1^ speed in the wind tunnel. In light of this, the difference between the CL_*r*_ for wheat and oat is probably larger than we estimated in this experiment as well (table 2).

Though CL_*r*_ values were significantly different between wheat and oat, the power law coefficient (*d*) from the raw curve fit (video-estimated stem curvature) along the windward edge was not, although *d* was only slightly higher in oat. This result indicates a relatively linear stem curvature at or near the reference deformation for oat as compared with wheat. The modelled curvature at the reference deformation of the stem (*d*) in the stem bending equation did not vary significantly among crops (*p* = 0.15), blocks (*p* = 0.68) or cultivars nested within crops (*p* = 0.09). Similarly, mean *d* coefficients among crops were not significantly differentiated at the time of CL_*r*_ and *C*_*d*_ estimation (table 2). The CL_*r*_ accounts for both drag force and stem curvature, with the following implication for *d* in this experiment: while insignificant stem curvature differences (*d*) were detected from the video data alone, considering the transit of the midpoint along the bending stem under a known force via CL_*r*_ revealed that wheat stems could significantly resist more of the drag force from a more arced stem.

**Figure 4.**
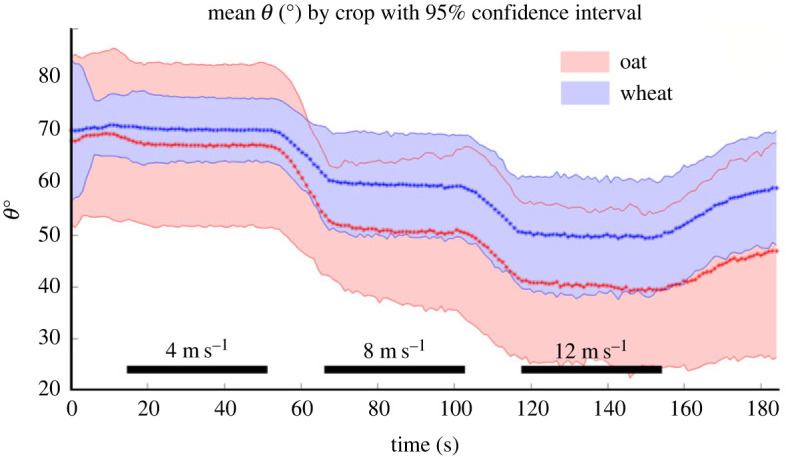
Observed stem bending angle values *θ* averaged for wheat and oat at each second over the course of the wind tunnel testing regime. Dotted lines indicate the mean *θ*; red for oat and blue for wheat. Shaded areas represent 95% confidence intervals around the mean *θ* value for each crop at a given second.

Along with a significantly lower CL_*r*_, oat reached a significantly lower bending angle at maximum velocity in the wind tunnel (*θ*_max_) compared with wheat and did not recover as much as wheat once the wind tunnel fan was shut off. The *θ*_max_ varied significantly by crop (*p* = 0.02), while block (*p* = 0.67) and cultivar nested within crop (*p* = 0.16) effects were not significant for *θ*_max_. Wheat had a significantly higher mean *θ*_max_ than oat (table 2). The averaged bending profiles for oat and wheat depicted by the bending angle *θ* over time are shown in figure 4. Wheat exhibits a significantly greater *θ* especially during the 12 m s^−1^ maximum speed in the wind tunnel. Recovery angle did not vary significantly among crops (*p* = 0.11) or cultivars nested within crops (*p* = 0.08), but did vary significantly among blocks (*p* = 0.03). This significant block effect for recovery angle implies that position on the greenhouse bench affects the ability of these cereals to bounce back following wind stress, possibly due to etiolation of stems in blocks positioned at the interior of the greenhouse bench. At the crop level, mean *θ* recovery angles were higher in wheat than oat, though not significantly (table 2). Further, the recovery angle was notably higher in wheat following the cessation of the 12 m s^−1^ maximum speed in the wind tunnel (figure 4).

*Phenotypic correlations*: Video derived plant phenotypes such as CL_*r*_ were significantly correlated with underlying botanical and physiological phenotypes. Correlations across cultivars of major video-derived phenotypes (CL_*r*_, *C*_*d*_, *θ*_max_, recovery angle and *d*) with plant physical traits are presented in table 3.

**Table 3.** Pearson correlation coefficients among phenotypes related to plant stem bending.

	1.	2.	3.	4.	5.	6.	7.	8.	9.
1. *C*_*d*_									
2. CL_*r*_	0.36								
3. *θ*_max_	−0.22	0.55*							
4. *d*	0.04	−0.29	0.15						
5. heading date	−0.15	−0.18	−0.21	−0.56*					
6. plant strength	0.44*	0.92^***^	0.41	−0.23	−0.01				
7. height	0.12	−0.60^***^	−0.54^**^	0.11	0.31	−0.44			
8. biomass	0.13	−0.39	−0.37	0.01	0.58^**^	−0.27	0.77^***^		
9. recovery angle	0.01	0.12	0.48*	0.48*	−0.35	0.34	−0.32	−0.38	

Significance codes: ‘***’ 0.001, ‘**’ 0.01, ‘*’ 0.05.

Parameters quantifying stem bending (such as CL_*r*_ and *θ*_max_) revealed both significant differences among crops and correlations with physiological traits. The positive relationship between stem strength and *θ*_max_ (table 3) indicates that a higher wind velocity was required to achieve the reference deformation across the experiment for stronger stemmed plants. Stronger cereal stems are more likely to remain upright under wind stress [35], which explains the positive correlation between stem strength and drag coefficient due to a stronger cereal’s ability to remain exposed to the airflow for increasing wind drag. The positive relationship between increased *θ*_max_ and CL_*r*_ also indicates that more upright plants resist a greater proportion of the drag force on the stems. The positive relationship between CL_*r*_ and lodging resistance in the field has been noted previously among oat varieties: varieties resisting greater torque exhibit lower lodging severity or lodging angles [36,42]. Furthermore the inverse relationship between CL_*r*_ and the modelled stem bending coefficient *d* confirms [36] findings, as more flexible cereal stems are capable of resisting greater torque from wind-induced drag.

The CL_*r*_ was highly sensitive to stem strength and height in this experiment. Cereals with higher CL_*r*_ values would also be expected to have a faster rate of bending recovery (measured in degrees per unit time) following wind exposure. This study confirms that trend, as wheat had the highest average recovery of 5.2° at the end of the deceleration from 12 m s^−1^ wind speed (table 3), which was greater (though not significantly so) than oat. Strength derived from stem curvature is optimal, as it distributes the strain along the entire stem and not at the base of the cereal, thereby avoiding plastic deformation and increasing both stem and root lodging resistance [36].

### 3.2. Deformation and failure profiles of wheat and oat from simulations

From the wind tunnel experiments, we conclude that the phenotypes—the coefficient of lodging resistance CL_*r*_, plant height, biomass and bending angle at maximum velocity—predict the stem strength behaviour and differences between the wheat and oat stems. However, we cannot confidently determine the causal relationship of this observed behaviour and phenotypes with the physical traits of the plants such as chemical composition (lignin, hemicellulose and cellulose content), tissue characteristics (volume fractions and cell structure of parenchyma, bundles, etc.) and plant morphology (internode height, cross-section details). Adding each of these traits in the experimental study will lead to exorbitant labour and resource costs, which poses substantial limitations. By contrast, our comprehensive simulation framework presents an opportunity for a physics-based understanding of the origin of experimentally observed behaviour and its quantitative relation with physical traits at different length scales.

**Figure 5.**
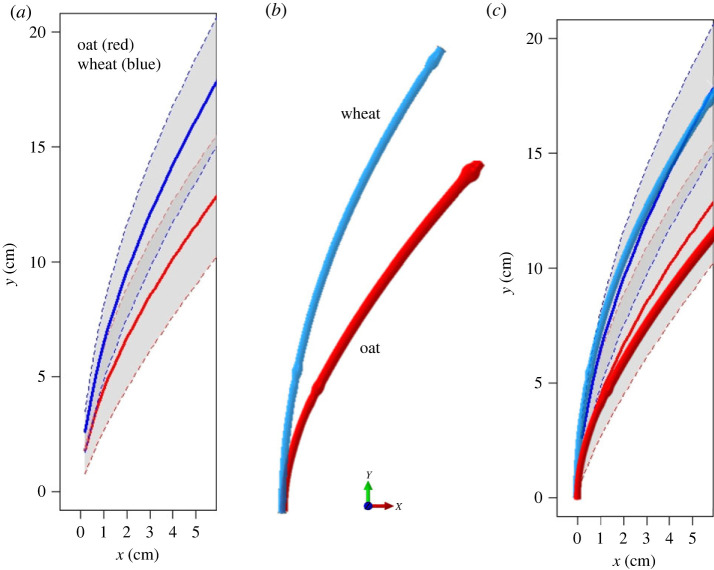
(*a*) Observed stem deformation in wind tunnel under wind velocity *v*_*w*_ = 8 m s^−1^, (*b*) simulated stem deformation for *v*_*w*_ = 8 m s^−1^ and (*c*) superposition of (*a*) and (*b*).

Before tracing the origin of lodging behaviour via our framework, we validate it by comparing the simulated deformation profile with the experimentally observed one in the wind tunnel (figure 5). We extracted the deformation profile from wind tunnel video frames in the time interval when the flow velocity was stable at 8 m s^−1^ for all oat and wheat cultivar/replicate combinations. Data were converted into the mean deformation profiles with 95% confidence intervals for both cereals (figure 5*a*). All the required parameters are listed in the electronic supplementary material, text S2 except the drag coefficient for the panicle *C*_*d*,*p*_ in equation (2.4) for simulating the deformation profile under a given wind velocity. In general, the drag coefficient is not an absolute constant for a given deformable body. The drag coefficient varies with the wind velocity and transient shape characteristics. We assume a *C*_*d*,*p*_ = 0.5, which is close to the drag coefficient of a vertical cylinder in cross flow and an acceptable simplification for the panicle geometry. The simulated deformation profiles for the wind velocity *v*_*w*_ = 8 m s^−1^ are superimposed on the observed deformation profiles in figure 5*c*. The comparison demonstrates that the simulations predict sufficiently well the observed deformation profiles under the given wind conditions. The measured phenotypes in wind tunnel including CL_*r*_, *θ* and *d* are essentially derived from the deformed stem profile.

**Figure 6.**
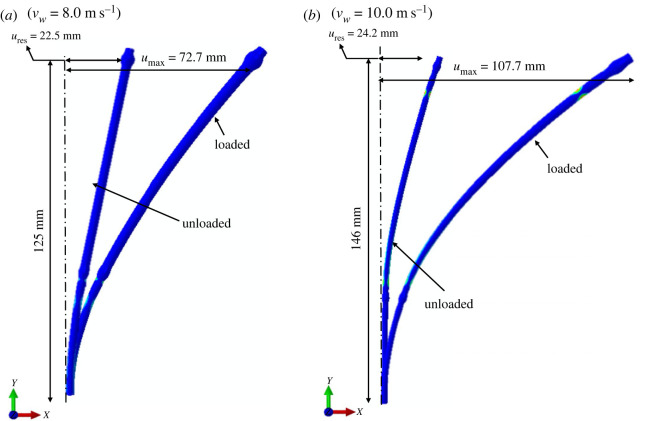
Simulated deformed profiles for both (*a*) oat and (*b*) wheat after loading/unloading steps at limiting wind velocity levels 8.0 and 10.0 m s^−1^, respectively.

Next, we investigate the failure behaviour of both wheat and oat stems, focusing on the elastic and elastoplastic ranges of stems’ mechanical response under the wind loads. The elastic response of stems means its ability to resist deformations caused by external wind forces and to return to its original position when the force is removed. In the elastic range, the plant stem tissues experience no material damage. On the contrary, in the elastoplastic response, plant tissues are permanently damaged and stems fail to fully recover to their original position after the applied force is removed. To this end, we simulate multiple scenarios for wind-induced drag force levels corresponding to wind velocity *v*_*w*_ experienced in the wind tunnel. As mentioned, the simulations are performed in two steps: the *loading step* and *unloading step*. Variables *u*_max_ and *u*_res_ are introduced as the maximum absolute values of the displacement of the upper node of the models after loading and unloading steps, respectively (figure 6). If the stem recovers to its initial position, then *u*_res_ = 0, and the stem remains in the elastic range. Whereas, a non-zero residual displacement after unloading (*u*_res_ ≠ 0) implies the elastoplastic behaviour of the stem with permanent material damage.

**Figure 7.**
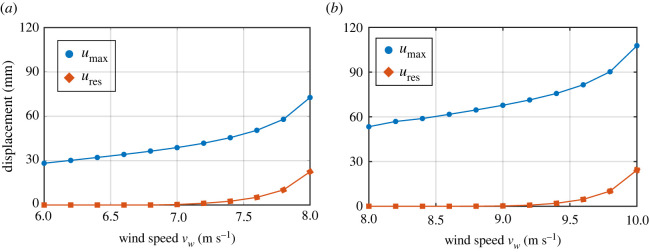
Simulated *u*_max_ and *u*_res_ with wind velocity *v*_*w*_ for (*a*) oat and (*b*) wheat. *u*_max_ and *u*_res_ are defined as the maximum absolute values of the displacement of the upper node after loading and unloading steps.

**Figure 8.**
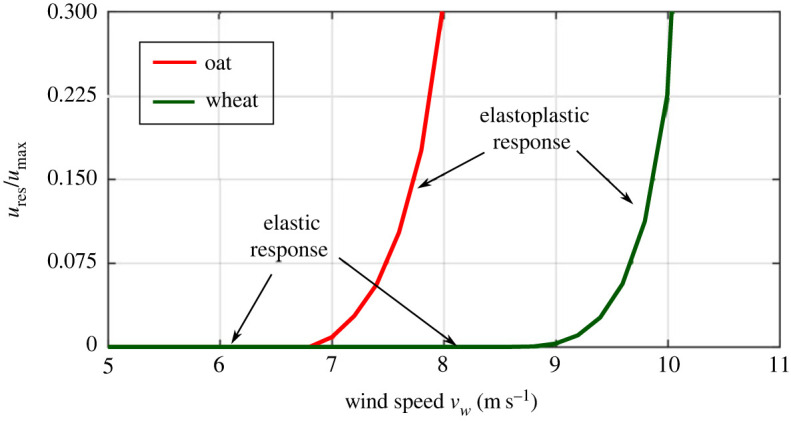
Residual ratio *u*_res_/*u*_max_ versus wind velocity *v*_*w*_ for oat and wheat simulations with distinct elastic and elastoplastic regions.

Figures 6–8 illustrate the relative differences in the failure behaviour of wheat and oat stems. We define a residual ratio corresponding to wind velocity *v*_*w*_ as *u*_res_ scaled with respect to *u*_max_, that is *u*_res_/*u*_max_. For both stems, we simulated wind velocity levels until the residual ratio *u*_res_/*u*_max_ reaches 0.3, which we assume as the maximum wind-induced drag force endured by the stems before failure. This limiting wind velocity level is 8.0 m s^−1^ for oat and 10.0 m s^−1^ for wheat. Figure 6 shows deformation profiles for both cereals after loading/unloading steps for these wind velocity levels with *u*_res_ and *u*_max_ values. Figure 7 shows the absolute values of *u*_max_ and *u*_res_ with relevant wind velocity range for oat and wheat. Figure 8 plots the residual ratio *u*_res_/*u*_max_, with applied wind velocities *v*_*w*_. The figure also displays two distinct regions denoting the elastic and elastoplastic responses of stems against the applied wind load. This plot can also be interpreted as a representative load–displacement behaviour. From these figures, it is clear that the wheat stem fails at a higher wind velocity level compared with oat. Moreover, the recovery at the same level of wind velocity is greater for wheat than oat. Our simulations of *u*_res_ (figure 7) and *u*_res_/*u*_max_ (figure 8) agree with the experimentally measured bending angle summarized in figure 4. The oat plants experienced permanent damage during 8 m s^−1^ wind velocity regime with little recovery after the wind tunnel is switched off, which is close to the simulated limited value of 8.0 m s^−1^. By contrast, wheat plants did not show any visible damage with relatively greater recovery after withstanding maximum wind velocity of 12 m s^−1^. Our simulations successfully predict these differences.

### 3.3. Simulations derived insights into the origin of stem deformation and failure behaviour

The finite-element simulations show excellent agreement with the deformation profiles observed in the wind tunnel experiments. From both experiments and simulations, we establish two important conclusions defining stem mechanical behaviour of wheat and oat. These conclusions are (i) wheat deformation response is stiffer than the oat, and, therefore, the stem strength of wheat is higher than the oat and (ii) the ability to recover the original configuration after the wind exposure is greater in wheat compared with oat. The integrated multi-scale material model presents an opportunity to explain the origin of overall stiffer response of wheat that leads to its lodging resistance, which we explain in the following discussion.

A careful investigation of parameters characterizing the hierarchical structure of oat and wheat reveals two important differences: (i) the lignin percentage in the cell wall material in wheat is higher than oat (electronic supplementary material, table S1) and (ii) the cell wall fraction in parenchyma tissues of wheat stems is higher than that of oat stems (electronic supplementary material, table S2). These differences lead to the stiffer and stronger soft-pith region in wheat as compared with oat, which is evident from the calculated stiffness and strength parameters in electronic supplementary material, text S2. In particular, the transverse stiffness and strength (*E*_1_, *E*_2_ and *σ*_11_, *σ*_22_) are substantially higher in wheat than oat. The other important distinction in the plant morphology of both cereals is that the first internode cross-section in wheat is almost solid, with no hollow space (electronic supplementary material, table S3). The shear force and moments generated by wind and gravity forces are higher in the lower part of the plant. The stronger solid-pith in wheat provided an overall stiffer response.

**Figure 9.**
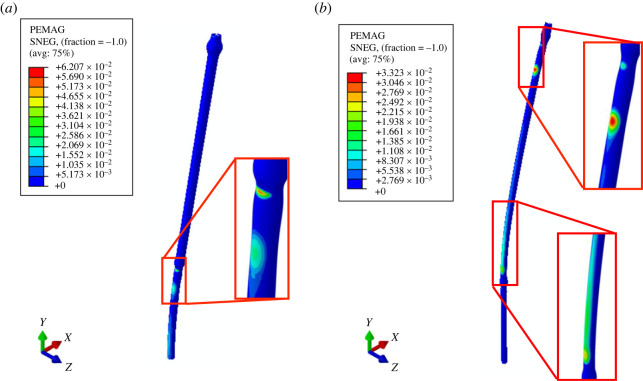
Distribution of plastic strain (PEMAG) among simulated (*a*) oat’s stem after unloading step (*v*_*w*_ = 8.0 m s^−1^) and (*b*) wheat’s stem after unloading step (*v*_*w*_ = 10.0 m s^−1^).

The recovery of cereals to the original position following wind exposure is a critical component of lodging resistance. The amount of recovery relates to the extent of material damage at the tissue level. Cereals, and plants in general, have the ability to heal tissues after the damage. Therefore, if the material damage is limited, then the stem heals to the original strength, contributing towards its lodging resistance. Plastic strain magnitude profiles (PEMAG) shown in figure 9 represent the simulated extent of material damage in both wheat and oat. The nodal region near the first internode is particularly important. In the oat stem, the plastic strains are concentrated in the region just below the first node, potentially contributing to lower recovery angles in oat versus wheat within the presented wind tunnel study. However, in the wheat stems the plastic strains are distributed along the entire first internode region, avoiding any significant localized material damage. This optimal distribution in wheat results in better recovery, and, hence, better lodging resistance as compared with oat.

**Figure 10.**
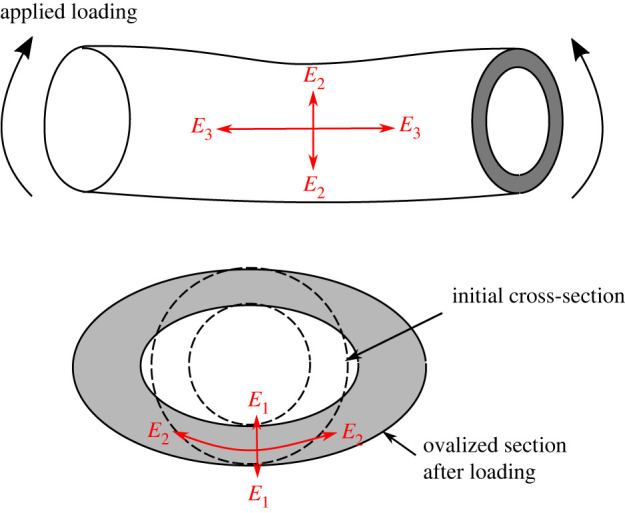
Example ovalization of cross-section with associated stiffness parameters.

The origin of these distinct mechanisms in oat and wheat lies in the underlying physics at different length scales in its hierarchical organization. The strength of both soft-pith and outer-shell regions is determined in part by the concentration of lignin in the cell wall material [22,43]. Higher parenchyma cell wall fraction and lignin percentage in wheat results in approximately three times transverse stiffness and strength parameters in comparison with oats. The localized material damage is initiated by the ovalization of plants' cross-section. Figure 10 visually explains this ovalization process of stem cross-section with the associated stiffness parameters. With comparable axial stiffness *E*_3_, the transverse stiffness (*E*_1_ = *E*_2_) of solid-pith dictates the ovalization induced material damage. Thus, the reduced transverse stiffness and strength of soft-pith region in oat facilitate the ovalization with limited prevention to local material damage, as compared with wheat.

**Figure 11.**
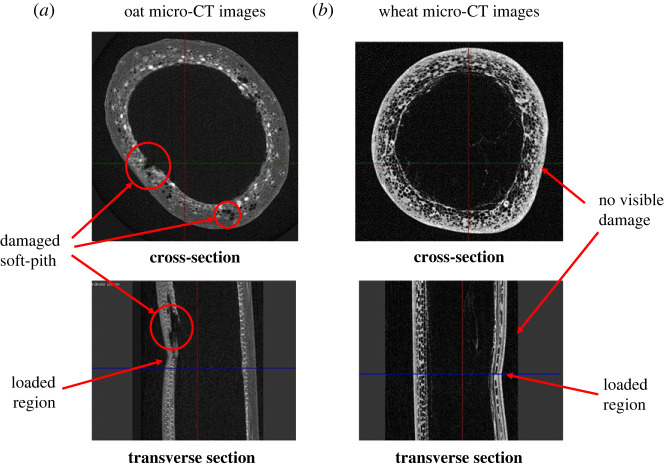
Micro-CT observations into the (*a*) oat and (*b*) wheat samples after loading/unloading process, demonstrating evident cross-section ovalization and material damage in oat, as compared with wheat. Images provided by MDRCBB.

These insights are also supported by the micro-CT investigations into the stem samples after loading/unloading process (see figure 11). Oat and wheat internode samples were subjected to displacement-controlled loading using a universal testing frame (MTS Instron 858 Mini Bionix II) with a 500 N load cell. Once the specimen loses structural integrity, the force begins to decrease. Thereafter, we unloaded the specimen and imaged the loaded area using a micro-CT machine (XT H 225, Nikon Metrology Inc., Brighton, MI, USA). Figure 11*a* shows a clear ovalization of oat cross-section, as compared with wheat. A closer look at these images reveals ruptured tissues in the oat cross-section and transverse section views at multiple places, while wheat images show intact cross-section with no visible damage to the soft-pith material. This visual evidence strongly supports our model-derived insights into the failure behaviour of both cereals.

### 3.4. Limitations of the present study

We acknowledge that the single plant experiments presented here do not reproduce precisely the aerodynamic loads occurring in field conditions, where plants are organized into a canopy [44–46] and are exposed to strong wind gusts during storms. Cereal crops in nature, thus, experience the wide range of scales induced by surface layer turbulence [47] and wind–canopy interactions characterized by mixing layer instabilities resulting in spatially coherent oscillations of multiple stems [14,48,49]. Hence, under field conditions, the effective frontal area, the drag coefficient and the incoming flow velocity vary constantly, all contributing to significant temporal variability of the aerodynamic loads and of the resulting drag force. In summary, the drag coefficient *per se* is not an ideal plant quantity to estimate and report, since its quantification depends on the plant deformation, applied wind flow Reynolds number, and of course its geometrical characteristics. Therefore, drag coefficients from different studies with varying experimental conditions should not be compared directly. Nevertheless in our specific case the drag coefficient at reference deformation is a valuable check-point combining independent measurements of different variables including wind forcing, plant geometry and dynamic response. Hence, we report it, acknowledging its limited role.

We emphasize that we have only a measured force for a specific stem deformation in addition to the limitation to assess the frontal area. In the absence of direct measurement of the frontal area, the lower bound estimates of drag coefficient may have excluded aerodynamic characteristics of crops such as reorientation of leaf foliage in the wind direction. Moreover, the samples were grown in the greenhouse, and we did not study the effect of greenhouse environment on the chosen cultivars. Other *in situ* factors such as turgidity were not measured, which possibly contributed to the overall aerodynamical and mechanical behaviour of wheat and oat. Therefore, conclusions from phenotypes such as drag coefficient cannot be surely extended to field conditions.

This study shows that multi-scale model-based predictions of cereal stem bending fall within the stem bending observations of independently assessed cereals in a wind tunnel. An important limitation of this study is that the multi-scale models were validated observationally against a set of replicated oat and wheat cultivars representing varying degrees of field lodging susceptibility. These tested cultivars both included and went beyond the single oat and wheat cultivars from which the input anatomical data for the multi-scale models were derived. Though, the oat and wheat multi-scale models predict stem bending patterns that fit within confidence intervals of respective observations, they cannot predict individual cultivar outcomes with confidence in this relatively small study. We also anticipate that the stem bending behaviour of cultivars grown in field may differ from the same cultivar grown in a greenhouse environment. Nonetheless, the aggregate differences between oat and wheat are able to be confidently predicted, and bode well for using multi-scale modelling approaches to elucidate structural mechanisms to improve lodging resistance targeting specific cultivars in the future.

As the stem bending response reveals an elastic behaviour, advanced studies of crop lodging may require a structural dynamic approach based on the comparison between the natural frequency of the stem and the forcing frequency of the turbulent flow in the canopy mixing layer [45,50]. Additional loading due to rainfall on the plant may contribute to lodging during severe weather [51]. In the case of direct wind exposure, e.g. experienced by the windward rows of cereals in a field, our experiments are, however, representative, and ensure well-controlled boundary conditions to study stem deformation under prescribed wind conditions. In this respect, the reference deformation defined here, corresponding to 50° stem angle from the ground, represents a threshold at which root and or stem lodging probably occurs in the field induced by natural wind. Therefore, the average drag coefficient for the tested cereals determined in these experiments can be assumed to represent aerodynamic loads on stems under direct wind stress (windward rows), while the estimated coefficient of lodging resistance can be assumed to represent the crop behaviour under a known force, regardless of the specific wind flow or canopy arrangement conditions.

## 4. Conclusion and outlook

In this article, we established that the multi-scale model-based finite-element simulations in combination with the controlled wind tunnel experiments provide important insights on the lodging behaviour and its relation with the physical traits of the plant. The experimental observations supported the conclusion that the wheat stems' response is stiffer than the oat. These conclusions were in agreement with the relatively better lodging resistance of wheat as compared with oat. However, these experimental observations did not in themselves establish causal relationships of this observed behaviour with the physical traits of the plants. Our simulation framework comprehensively includes chemical composition (lignin, hemicellulose and cellulose content), tissue characteristics (volume fractions and cell structure of parenchyma, bundles, etc.) and plant morphology (internode height, cross-section details). This feature of our simulation framework directed us towards the microstructure origin of the observed macroscopic stem bending response. We deduced that higher parenchyma cell wall fraction and lignin percentage in wheat result in better resistance against material damage through ovalization, explaining better stem strength of wheat as compared with oat. While the predictive ability of our model was tested against wind tunnel observations under minimized environmental and temporal variations, its extension to more realistic settings can be implemented through the coupling with turbulent canopy models accounting for unsteady forces and moments.

This article also outlines a unique opportunity presented by collaborative efforts of researchers from mechanics and simulation background with plant scientists to address complex problems influenced by plant, agronomic and environmental characteristics. Computational modelling and the wind tunnel experiment highlighted a suite of traits and possible interactions at the crop level (such as CL_*r*_ and solid-pith variation) in oat versus wheat that would not have been identified using the individual approaches. For the complete understanding of lodging behaviour of cereals, expertise from solid mechanics, finite-element analysis, experimental fluid dynamics in conjunction with plant scientists is required. More importantly, the validated finite-element analysis model that predicted observed stem bending differences in oat and wheat could be used to manipulate simulated traits to identify breeding targets before breeding efforts begin.

## Data Availability

All videos collected in the wind tunnel are available from a University of Minnesota Libraries DRUM repository: https://doi.org/10.13020/sat9-xx53 [52]. The readme file at the GitHub repository https://github.com/Hortus/windtunnel_video_analysis/blob/master/README.md details the scripts used and location of the data analysed from the wind tunnel experiment. Multi-scale material model codes in Python, Abaqus model file and python script for automatized simulations at different wind speed levels pertaining to multi-scale finite-element model simulations are available from the Dryad Digital Repository: https://doi.org/10.5061/dryad.612jm644j [53]. Supplementary material is available online [54].
